# Synthetic biodegradable polymer materials in the repair of tumor-associated bone defects

**DOI:** 10.3389/fbioe.2023.1096525

**Published:** 2023-02-16

**Authors:** Honghao Yu, Haifeng Liu, Yuan Shen, Qiang Ao

**Affiliations:** ^1^ Departments of Spine Surgery, Shengjing Hospital of China Medical University, Shenyang, China; ^2^ Department of Tissue Engineering, China Medical University, Shenyang, China; ^3^ Departments of Neurosurgery, Shengjing Hospital of China Medical University, Shenyang, China; ^4^ NMPA Key Laboratory for Quality Research and Control of Tissue Regenerative Biomaterial and Institute of Regulatory Science for Medical Device and National Engineering Research Center for Biomaterials, Sichuan University, Chengdu, China

**Keywords:** synthetic, degradable, polymer materials, bone defect, repair

## Abstract

The repair and reconstruction of bone defects and the inhibition of local tumor recurrence are two common problems in bone surgery. The rapid development of biomedicine, clinical medicine, and material science has promoted the research and development of synthetic degradable polymer anti-tumor bone repair materials. Compared with natural polymer materials, synthetic polymer materials have machinable mechanical properties, highly controllable degradation properties, and uniform structure, which has attracted more attention from researchers. In addition, adopting new technologies is an effective strategy for developing new bone repair materials. The application of nanotechnology, 3D printing technology, and genetic engineering technology is beneficial to modify the performance of materials. Photothermal therapy, magnetothermal therapy, and anti-tumor drug delivery may provide new directions for the research and development of anti-tumor bone repair materials. This review focuses on recent advances in synthetic biodegradable polymer bone repair materials and their antitumor properties.

## Graphical Abstract

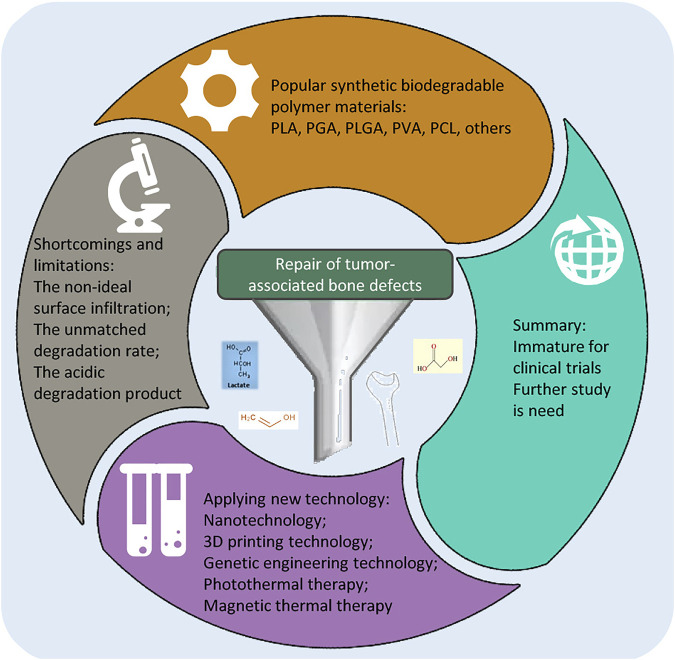

## 1 Introduction

Bone tumors and cancer metastasis to bone pose a serious threat to human health, especially osteosarcoma being the most common. For example, the age-standardized incidence rates for 0–79 ranged from 2 cases per million in Southern Asia to 4.2 in Sub-Saharan Africa, and the 5-year survival rate of osteosarcoma treated by amputation surgery has been 5%–20% for decades. The standard clinical treatment strategy for bone cancer involves surgical resection and reconstruction of the involved bone followed by adjuvant radiotherapy or chemotherapy. Surgical resection of bone malignancies can cause bone defects or delayed bone healing, severely affecting the patient’s quality of life (Isakoff et al., 2015; Rojas et al., 2021; Chen and Yao, 2022). Finding the ideal repair materials has always been a challenge for orthopedic surgeons. According to the different components of the materials, synthetic bone repair materials can be mainly divided into metal materials, bioceramics, calcium phosphate bone cement, polymer materials, composite materials, tissue engineering materials, *etc.* Among them, polymer materials have been used as bone-filling materials since the mid-1960s, with the advantages of immunocompatibility and good biocompatibility (Cao et al., 2021). Polymer materials commonly used in bone tissue engineering research can be divided into natural polymer materials and synthetic polymer materials. Among them, natural polymers include collagen, fibrin, chitin, hyaluronic acid, sodium alginate, chitosan, etc (Zhang et al., 2021; Zhang et al., 2022). Synthetic polymers include polylactic acid (PLA), polyglycolic acid (PGA), poly (lactic-co-glycolic acid) (PLGA), Polyvinyl alcohol (PVA), polycaprolactone (PCL), polyamide and other synthetic polymers (He et al., 2018). Natural polymer materials have good biocompatibility and thus contribute to improving cellular properties. However, they are difficult to design, with limited processing capacity, high contamination risk, and instability and variability of the property. Compared to natural polymer materials, synthetic polymer materials have stable chemical properties, and can be modified to obtain specific properties. Other advantages of synthetic polymer materials include cost-effectiveness, mass-production capacity, and longer storage time (Dwivedi et al., 2020). Synthetic polymer materials mainly exist in the form of scaffolds in bone repair, and composite materials with tissue cells, growth factors, and other materials can improve the biological activity and biocompatibility of the material itself (Yassin et al., 2017). The development of biomaterials provides broad prospects for the future treatment of bone tumors, and worldwide scholars constantly explore biomaterials that can both repair bone defects and inhibit tumor recurrence. This review focuses on new advances in synthetic biodegradable polymer bone repair materials and their antitumor properties.

## 2 Polymer materials containing polylactic acid, polyglycolic acid or poly (lactic-co-glycolic acid)

PLA, PGA, and PLGA are currently the most widely used extracellular matrix materials for bone tissue engineering. PLA is hydrolyzed *in vivo* to produce lactic acid, and PGA is degraded to hydroxyacetic acid *in vivo*, which can easily participate in the metabolism *in vivo* (Liu and Yu, 2021). PLA and PGA have good biocompatibility, good mechanical properties, and plasticity, which have been approved by the US Food and Drug Administration (FDA) for extensive clinical application. However, these materials have defects such as poor hydrophilic nature and fast degradation rate, and the intermediate products will accumulate in a local range, resulting in low pH values. The effect of the two materials directly used to repair bone defects is not ideal, and other materials need to be added for performance optimization (He et al., 2018). PLGA is also one of the most commonly used synthetic materials for bone defect repair and regeneration. It is a biopolymer material composed of random polymerization of two monomers, lactic acid and hydroxyacetic acid in different proportions. The presence of PGA makes the degradation rate of PLGA faster than PLA, and the degradation time of PLGA is prolonged as the proportion of propylene cross-esters in the copolymer composition increases. This synthetic polymer was first used for biomedical use in the early 1970s and has received extensive attention and research in bone tissue engineering for its good mechanical properties, controlled degradability, good biocompatibility, and excellent plasticity (Martins et al., 2018; Su et al., 2021). The unique physicochemical properties of the material give it broad biomedical applications, ranging from PLGA polymers for tissue regeneration scaffolds to drug delivery systems, and other applications for parenteral administration, diagnostic, basic, and clinical research, including cancer, cardiovascular disease, tissue engineering, and vaccines (Altındal and Gümüşderelioğlu, 2016; Ray et al., 2017). The synthesis method of PLGA can be divided into two categories: (1) direct polymerization of lactic acid and glycolic acid, which usually yields PLGA with low molecular mass and wide relative molecular mass; (2) ester open ring polymerization of propylene ester, which can obtain higher relative molecular mass and more uniform products (Zhao et al., 2021). There are many modification methods for PLGA scaffold, mainly for blending modification and surface modification. Mixed modification is made by mixing other substances, such as hydroxyapatite, tricalcium phosphate, magnesium hydroxide, and other inorganic substances, which can improve the mechanical properties and hydrophilicity of the scaffold, change cell behavior, and promote bone production. Surface modification is coated on the scaffold surface with a layer of active material that regulates interactions between the cell-scaffold material (Danhier et al., 2012; Rocha et al., 2022). The researchers found that physically mixing the right amount of hydroxyapatite and PLGA into a composite scaffold could improve the scaffold’s mechanical strength and that the hydroxyapatite would automerize on the scaffold surface to form a nanostructure, which facilitates cell adhesion and migration and promotes new bone generation. There are also studies to add magnetic nanoparticles to PLGA/hydroxyapatite scaffold, which can effectively inhibit and kill bone tumor cells by magnetic hyperthermia under the external magnetic field, and the addition of magnetic nanoparticles can enhance cell adhesion, proliferation, and differentiation (Li M. et al., 2019). Rong et al. made adriamycin-encapsulated PLGA nanoparticles in a porous nano-hydroxyapatite/collagen scaffold, ADM-PLGA-NHAC, and evaluated the performance of the vehicle scaffold using various techniques such as scanning electron microscopy and *in vitro* sustained release. The results of the *in vitro* tumor-suppressor experiments showed that the ADM-PLGA-NHAC scaffold extract had a strong anti-tumor effect on the MG-63 osteosarcoma cells (Rong et al., 2016). Wang et al. successfully built the bioglass molybdenum disulfide complex (BGM) by fixing the molybdenum disulfide polylactic acid glycolic acid (MoS_2_-PLGA) membrane on the surface of the 3D printing bioactive glass bracket. Moreover, they demonstrated the inhibitory effect of this complex on tumor cells *in vitro* and *in vivo* trials (Wang et al., 2020). Bone infection is a serious complication after bone defect repair, and it is often difficult to achieve effective concentrations at the infection site, and the PLGA stent loaded with antibiotics can release high concentrations of antibiotics locally in bone repair to achieve therapeutic purposes. Researchers by studying the antibiotic release curve found that antibiotics in the first few hours of explosive release, then sustained low dose slow release, which is crucial to repair infectious bone defects, early local microenvironment of high concentration of antibiotics to completely kill bacteria, sustained low doses of antibiotics help to inhibit the growth of bacteria (Gao et al., 2016; Aragón et al., 2019). PLGA has shown great potential in drug delivery and tissue engineering, and its application in bone defect repair after tumor resection may become a new choice for orthopedic doctors.

## 3 Polymer materials containing polyvinyl alcohol

PVA is a linear synthetic polymer with biocompatibility, biodegradability, and chemical stability. PVA is often used in bone repair materials as a component of composite materials to improve the mechanical strength, hydrophilicity, and cell compatibility of scaffold materials (Pourjavadi et al., 2020). Chen et al. prepared the PVA/β-tricalcium phosphate composite scaffolds by melting deposition formation, and with the addition of β-tricalcium phosphate, the maximum stress of the scaffold reached 10.7 kPa, which significantly improved the carrying capacity of the composite scaffold. Chen et al. also verified the cell compatibility of the composite scaffold, and the results showed that the composite scaffold did not inhibit cell growth (Chen et al., 2019). Kaur et al. prepared PVA scaffolds loaded with different concentrations of graphene nanosheets by freeze-drying method. The addition of graphene nanosheets significantly improved the tensile strength of the polyvinyl alcohol scaffold. When the mass fraction of nanographene sheets was 1%, osteoblasts proliferated and differentiated best in the composite scaffold (Kaur et al., 2017). Xia et al. prepared PVA/silica hybrid fiber by electrospinning method. After 3 days of immersion in the simulated body fluid, layered apatite precipitation appeared on the surface of the hybrid fiber, so it is supposed that the fiber has certain bone inducibility conducive to bone repair (Xia et al., 2018). Lan et al. fabricated a well-developed porous carbon nanotube (CNT) reinforced polyvinyl alcohol/biphasic calcium phosphate (PVA/BCP) scaffold by a freeze-thawing and freeze-drying method. The degradation analysis indicated that the degradation ratio of scaffolds can be varied by changing the concentrations of BCP powders and CNTs (Lan et al., 2019). The study of Li Yao et al. determined the ratio of chitosan/polylactic lactate/hydroxyapatite/PVA composite, optimized the mechanical properties of bone scaffold, and the optimal ratio of the composite stent has high porosity and good mechanical properties (YM et al., 2022). The results of Istikharoh et al. demonstrated that polylactic ate glycolic acid/PVA coated hydroxyapatite nanoparticle composites have an optimal aperture, morphology, and degradability, showing their great potential as an effective bone scaffold for the repair of alveolar defects after tooth extraction (Istikharoh et al., 2020).

## 4 Polymer materials containing PCL

PCL has good degradability, superior biocompatibility, and strong mechanical properties, and it was approved by the FDA in the 1990s. PCL is a polyester organic polymer made by artificial synthesis. At physiological temperatures, the semi-crystalline PCL attains a rubbery state resulting in its high strength, high toughness, and excellent mechanical properties, along with good biocompatibility, slow degradation rate, and strong crystallinity. In addition to being biocompatible and biodegradable, PCL polyester is widely used as a scaffold for absorbable sutures, regenerative therapies, and drug delivery applications for its easy availability and cost-effectiveness, especially for building long-term implant delivery devices. Its longer degradation time makes it widely used to replace hard tissues, and load-bearing tissues by increasing their stiffness, and to replace soft tissues by reducing their molecular weight and degradation time (Dwivedi et al., 2020). In order to make the scaffold has antibacterial properties, Felice et al. prepared mixed with zinc oxide scaffold, the results show that high zinc oxide concentration can induce early mineralization, and adjusting the concentration of zinc oxide and the distribution in the material can realize the regulation of scaffold degradation rate, and the stent has antibacterial effect for S.*aureus* (Felice et al., 2018). Nanoparticles were cultured on a polydopamine-coated polyhexylactone scaffold and were significantly enhanced by the presence of a polydopamine coating by Lee et al. As nanoparticles, this scaffold was found to have good osteogenic activity *in vivo* experiments, which is expected to provide new material options for bony defect repair and regeneration (Lee et al., 2018). Wu et al. made the 3D-printed calcium silicate, PCL, and acellular extracellular matrix scaffolds, and observed that they exhibited excellent biocompatibility, cell adhesion, proliferation, and differentiation by increasing the expression of osteogenesis-related genes (Wu Y. A. et al., 2019). The microporous PCL scaffold matrix found by Palama et al. supports attachment, proliferation, and osteogenic differentiation of osteoblasts, whereas the polyelectrolyte multilayer fixation on the endopore surface maintains local dexamethasone release. These microporous scaffolds demonstrate the ability to treat dexamethasone as a local tumor therapy, and to promote the proliferation and differentiation of osteoblast-like cells *in vitro* (Palamà et al., 2017).

## 5 Other polymer materials

In addition to the above, there are some new materials, that researchers have brought them to many scholars through in-depth experiments, and may become candidate substitutes for bone defects after tumor resection after more long-term exploration. The applications of emerging technologies such as 3D printing, photothermal effects, and magnetic materials have gradually attracted the attention of researchers (Choi et al., 2017; Lu et al., 2018; Yang et al., 2018). Ma et al. studied an acrylic ester-based composite of polyfumarate as a potential clinical bone repair material with low heat release and suitable mechanical properties, which shows good biocompatibility. Furthermore, the surface morphology and hydrophilic properties can be adjusted by regulating the content of β-calcium phosphate, and maybe a promising bone repair material (Ma et al., 2019). Dang et al. combined 3D printing technology with a solvent thermal method to successfully synthesize CuFeSe_2_ nanocrystalline bioactive glass scaffold, which was verified to remove bone tumor cells *in vitro* by photothermal effects, and confirmed that the scaffold can kill Saos-2 tumor cells *in vivo* trials (Dang et al., 2018).

Using a strategy of combining 3D printing technology with photothermal properties for *in situ* ablation, Ma et al. made a 3D-printed calcium phosphate composite scaffold and modified it with graphene oxide to transfer the infrared laser energy to the photothermal effect (Ma et al., 2016). Zhang et al. prepared a hydrogenation black titanium dioxide coating with a micro/nano graded morphology by using the induction suspension plasma spraying technology. Good and controlled tumor growth inhibition by 808 nm near-infrared laser irradiation *in vitro* and *in vivo* (Zhang et al., 2019). Mondal et al. synthesized iron oxide, hydroxyapatite, and hydroxyapatite-coated iron oxide nanoparticles, and *in vitro* magnetic hyperthermia studies showed excellent thermal efficacy against MG-63 osteosarcoma cells, killing almost all experimental MG-63 osteosarcoma cells within 30 min of exposure (Mondal et al., 2017). KamitakaharaM et al. have prepared micron-scale magnetic nanoparticles with porous HA particles acting as a scaffold for bone regeneration, and the magnetic nanoparticles generate enough heat to kill tumor cells in an alternating magnetic field (Kamitakahara et al., 2016). Asa et al. have synthesized multifunctional magnetic ZnFe2O4-hydroxyapatite nanoparticles for local anticancer drug delivery and bacterial infection inhibition, and their study has found that drug-borne nanoparticles have the ability to inhibit the proliferation and growth of cancer cells. With the increasing concentration of the nanoparticles, the G292 cancer cell proliferation was inhibited, while the HEK normal cell proliferation was stimulated (Asa et al., 2019). Here listed in Table 1 are the synthetic biodegradable polymer materials for bone defects.

**TABLE 1:** Synthetic biodegradable polymer materials for bone defects (Henslee et al., 2011; Reichert et al., 2012; Jensen et al., 2014; Subramanian et al., 2015; Pang et al., 2019; Ding et al., 2022).

Graft composition	Bone defect	Regeneration outcomes	Limitations	References
3D printed polycaprolactone/β-tricalcium phosphate composite	3 cm, sheep, tibial	Radiographic union; mechanical restoration	Slow graft; resorption	Reichert et al. (2012)
Solid poly (propylene fumarate) rod/porous sleeve with PLGA microparticle	5 mm, rat, femoral	Improved defect fixation by solid rod; improved bone formation	Regeneration impeded by solid rod; no union	Henslee et al. (2011)
Salicylic acid-based poly (anhydride-ester)/polycaprolactone membrane	5 mm, rat, femoral	Ectopic bone formation suppressed; long bone regeneration improved (no mechanical testing)	Poor graft mechanical property; long-term remodelling unclear	Subramanian et al. (2015)
3D printing polylactic acid polymer-bioactive glass loaded with bone cement	5–6 mm in diameter and 5–6 mm in depth, rabbit femoral	Radiographic union; mechanical restoration; osteogenic differentiation ability	Improved scaffold degradation rate	Ding et al. (2022)
Surface-modified functionalized polycaprolactone	10 mm in diameter and 10 mm in depth, porcine, skull	Radiographic union; mechanical restoration	Acceleration of scaffold degradation	Jensen et al. (2014)
polyvinyl alcohol-borosilicate gel hybrid scaffolds	3 mm in diameter and 5 mm in depth, rat, femoral	Radiographic union; mechanical restoration	Unclear degradation rate	Pang et al. (2019)

## 6 Strategies for the development of anti-tumor polymer materials

Modern bone repair materials mainly include polymer materials, tissue-engineered bone, and related derived composites. With the continuous progress of technology in medicine and related fields, the research and technical level of modern materials are more microscopic. New technology is an effective strategy to develop new bone repair materials, and nanotechnology, 3D printing technology, genetic engineering technology, photothermal therapy, and magnetic thermal therapy have opened up new prospects for the research of anti-tumor bone defect repair materials (Venkatesan and Kim, 2014; Atak et al., 2017; Xu et al., 2022). The application of 3D printing technology makes bone repair stent preparation more refined, and different morphology, porosity, and performance can be made by changing the printing parameters. With the development of 3D printing and computer technology, complex bracket materials are more accurately used for production. Researchers are further studying the three-dimensional structures of prints mixed with living cells, growth factors, or other biomaterials, and 3D printed drug carrier materials are also gaining much attention (Raja and Yun, 2016; Han et al., 2017). Li et al. synthesized a composite scaffold of nano-hydroxyapatite (nHA) and reduced graphene oxide (rGO) sheets fabricated by self-assembly and found that nHA-rGO scaffolds killed all but 8% of osteosarcoma cells (MG-63) under 808 nm near-infrared laser irradiation for 20 min *in vitro* (Li et al., 2018). In order to optimize and make up for the lack of simple stent material performance, the researchers added all kinds of polymer, trace elements, drugs, seeds, cells, and related cytokines, usually different materials, to improve the mechanical properties, degradation properties, biocompatibility, and osteogenic properties, optimize the drug or cytokine load capacity (Li H. et al., 2019).

The composite of multiple materials is an effective way to produce new material properties. The bone stent composed of a single raw material cannot fully meet the needs of bone defect repair. Through the composite of various raw materials, the researchers hope to create an artificial stent that can perfectly fit the needs of bone defect repair. Direct local delivery of anti-tumor agents loaded into stents of bone repair materials is also an effective strategy (Zhou et al., 2017; Qu et al., 2021). Recently, it has been found that HA can inhibit the proliferation and induce apoptosis of various cancer cells, including osteosarcoma, breast cancer, gastric cancer, and colon, and liver cancer cells, but this biomaterial does not inhibit the proliferation of normal cells (Han et al., 2014; Zhao et al., 2018; Wu H. et al., 2019). Qing et al. Using transmission electron microscopy to observe the ultrastructural changes of the 2 cells showed that HA-NPs have a selective effect on different cell types of cells: supporting the proliferation of normal osteocyte cells while causing the apoptosis of osteosarcoma cells (Qing et al., 2012). It has been shown that chitosan plays a tumor-suppressor role by inhibiting glycolysis and reducing glucose uptake and ATP levels in cells, but chitosan has no such effect on normal cells. Moreover, the tumor cell surface has more negative charge than the normal cell surface, and the chitosan is positively charged, thus inhibiting tumor growth and metastasis (Ghezini et al., 2008). Wang et al. used selenium-doped hydroxyapatite nanoparticles (Se-HANs), which could potentially fill the bone defect generated from bone tumor removal while killing residual tumor cells, as an example to study the mechanism by which selenium released from the lattice of Se-HANs induces apoptosis of bone cancer cells *in vitro* and inhibits the growth of bone tumors *in vivo*, finding that Se-HANs induced apoptosis of tumor cells by an inherent caspase-dependent apoptosis pathway synergistically orchestrated with the generation of reactive oxygen species (Wang et al., 2016). Lu et al. reported a polydopamine (PDA)-coated composite scaffold consisting of doxorubicin (DOX)-loaded lamellar hydroxyapatite (LHAp) and poly (lactic-co-glycolic acid) (PLGA) in an attempt to reach dual functions of tumor inhibition and bone repair (Lu et al., 2021). Hess et al. combined a calcium phosphate microsphere with a matrix scaffold and prepared three calcium phosphate/calcium alginate beads as drug carriers by ion gel droplet extrusion method, and combined it into the scaffold matrix by cryogen method, which successfully released cisplatin and doxorubicin in experiments and showed a significant killing effect on osteosarcoma MG-63 cells (Hess et al., 2017).

## 7 Shortcomings and limitations of synthetic biodegradable polymer materials

Although the artificial synthesis of biodegradable polymer materials has great potential in bone defect repair, some deficiencies and limitations still need to be discussed. Some synthetic polymers are hydrophobic materials, and their surface infiltration is not ideal, affecting the adhesion, proliferation, and differentiation of cells, and then affecting the performance of the material and the repair of bone tissue. While the degradation rate of some materials does not match the speed of bone growth; the degradation product of some polyester materials is acidic and not conducive to new bone regeneration. For example, the degradation of PLA does not depend on enzymes, but through the hydrolysis of the ester bonds. For polylactic acid, the lack of hydrophilic groups in its structure makes the surface of the material hydrophobic. Low hydrophilicity is not conducive to cell adhesion, proliferation, and differentiation. In addition, PLA could produce acidic degradation products like lactic acid. The accumulation of lactic acid cannot be metabolized within a short time and resulting in a pH as low as 3.0 within 4 weeks, this may also dissolve some bone components as well (Liu et al., 2013; Han et al., 2020; Li et al., 2022). The earlier used PCL scaffolds did not provide optimal mechanical properties and biocompatibility, thus attempting to mix PCL with natural or synthetic polymers or ceramics. For example, combining calcium phosphate-based ceramics, bioactive glasses, and polymers into the PCL can improve the biomaterials with better mechanical properties, controllable degradation rates, and enhanced biological activity (Yan et al., 2019). In a word, some synthetic degradable polymer materials may have unsatisfied mechanical properties and biocompatibility and even affect osteogenesis. These problems need to be paid attention to and properly solved.

## 8 Summary

In recent years, biological materials carrying drugs or biological materials itself has the characteristics of anti-tumor effect have become a new direction, many scholars of malignant bone tumor resection of biological materials after thorough study, but there is not fully meet the requirements of bone repair materials, with bone defect repair and anti-tumor multifunctional materials still have a long distance from clinical trials. With the deepening of the research on the mechanism of osteogenesis, the continuous development of biomaterials, and the progress of material science and technology, it is expected to develop the ideal materials that meet the requirements of human bone repair (He et al., 2018). As a bone repair material, the core design idea is that, by inserting a stent in the damaged bone tissue, this stent can maintain good mechanical properties for a long period of time to support the repair of the defective bone tissue, and can be naturally degraded and replaced by the new bone tissue. The ultimate goal of the bone tissue engineering scaffold is to make an effective repair of the bone tissue, so cytocompatibility and bone inducibility are problems that cannot be ignored. A synthetic polymer as a scaffold matrix material, on the one hand, we should give full play to its own biocompatibility and biodegradability, and other excellent properties; on the other hand, it should also make up for the shortcomings of unsatisfactory cell compatibility due to their own hydrophobicity. How to better use synthetic polymer materials to simulate the extracellular matrix structure to promote the repair of bone tissue, and the minimum of its negative impact on the human body after implantation are still the problems that scholars need to further study and solve in the future. Different materials have certain advantages and disadvantages, so it is necessary to make optimal choices combined with material characteristics in clinical application. As more and more new biomaterials-related basic and clinical research are developed, the problem of postoperative bone defects and tumor recurrence in bone tumor patients will be solved gradually.

## Abbreviations

PLA, polylactic acid; PGA, polyglycolic acid; PLGA, Poly (lactic-co-glycolic acid); PVA, Polyvinyl alcohol; PCL, polycaprolactone.
